# Endovascular treatment of a pulmonary artery pseudoaneurysm caused by Swan-Ganz catheter deployment in an anticoagulated patient

**DOI:** 10.1259/bjrcr.20150064

**Published:** 2015-07-29

**Authors:** A M Ierardi, G Xhepa, A M Musazzi, M De Chiara, C Beghi, G Carrafiello

**Affiliations:** ^1^ Interventional Radiology, Department of Radiology, Insubria University, Varese, Italy; ^2^ Cardiac Surgery Department, Department of Radiology, Insubria University, Varese, Italy

## Abstract

We present a case report of an anticoagulated 78-year old man presenting a pulmonary artery pseudoaneurysm following Swan-Ganz catheter deployment after an aortic valve and aortic root replacement. Diagnosis was established by cone beam CT angiography and catheter angiographyand embolisation was achieved via a combination of plug and glue. This case emphasises the importance of endovascular techniques in the management of iatrogenic pulmonary pseudoaneurysms and shows the benefit of using highly hemostatic polymeric agent in anticoagulated patients to obtain a rapid and effective occlusion.

## Summary

An anticoagulated 78-year-old patient presenting with a pulmonary artery pseudoaneurysm (PAP) following Swan–Ganz catheter deployment after aortic valve and aortic root replacement was referred to our institution, and interventional radiology endovascular embolization was proposed. A combination of plug and glue were used to achieve a complete embolization. The case presented confirmed the importance of endovascular techniques in the management of iatrogenic pulmonary pseudoaneurysms (PSAs) and shows the benefit of using a highly haemostatic polymeric agent in similar situations to obtain rapid and effective occlusion.

PAPs are uncommon and may be congenital or acquired. Most PAPs are acquired and are associated with cardiovascular disease. The other acquired causes of PAPs include infection, iatrogenic causes, trauma and neoplasm.^1^ Iatrogenic PAP is the most common, followed by trauma. The iatrogenic causes of PAP include complications of the pulmonary artery (PA), right cardiac catheterization, chest tube insertion and biopsies.^2,3^ Haemoptysis is the most frequently presenting sign, and it ranges from acute severe haemorrhage to incidental findings on radiograph, CT scan or MRI. Owing to the risk of PSA enlargement and rupture, which leads to death in approximately 50% of the patients, prompt therapy is required.^4,5^

Treatment of PAPs includes surgical ligation, wedge resection, lobectomy, angiographic embolization, endovascular stent graft placement, and watchful waiting.^6^ Transcatheter embolization with stainless steel coils, platinum coils or detachable balloons is a practical, effective and safe therapeutic option.^1,3^

Here, we present a case of symptomatic PAP after Swan–Ganz placement in a patient who underwent heart surgery and was treated percutaneously by deploying an amplatzer vascular plug (AVP) and transcatheter *N*-butyl cyanoacrylate (NBCA) injection.

## Clinical presentation

**Figure 1.**
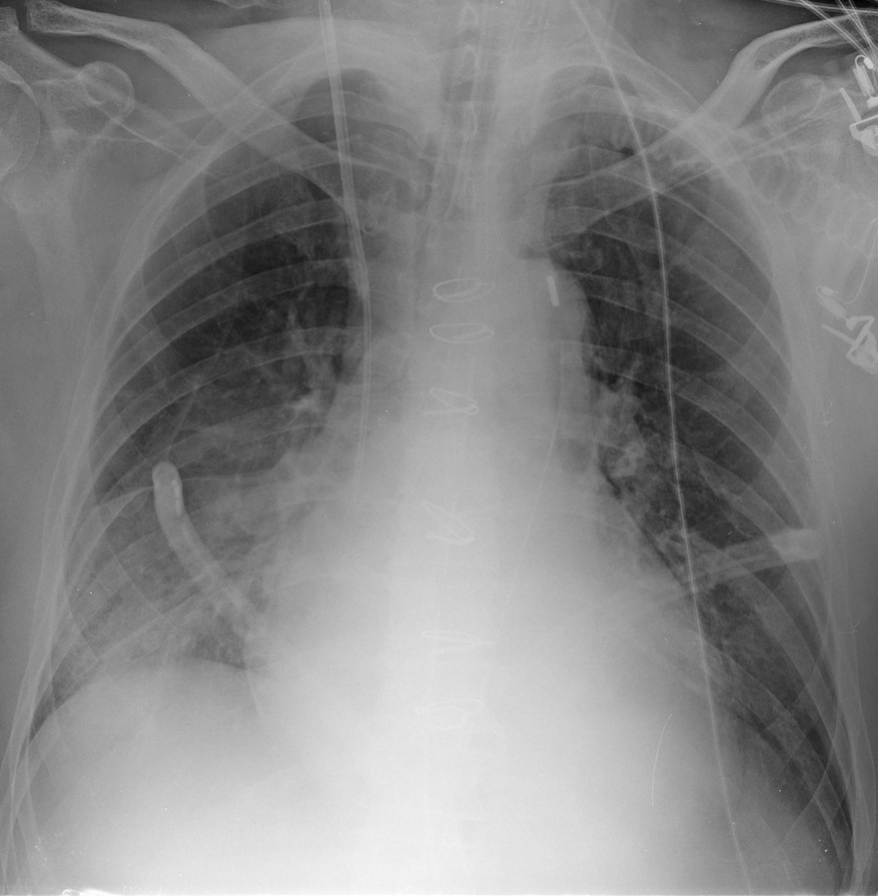
Chest radiograph performed in the recovery room showing a right basal pleural effusion.

**Figure 2.**
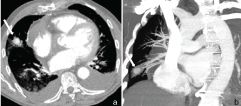
CT angiography showing the presence of a pulmonary artery pseudoaneurysm (arrow; a); maximum intensity projection reconstruction confirms arterial feeding (arrow; b).

A 78-year-old male with a history of dyspnea (New York Heart Association Stage III) was transferred to our Cardiac Surgery Department. Two- and three-dimensional echocardiography confirmed the presence of severe aortic valve stenosis and moderate valve regurgitation. The valve cusps appeared to be calcified with restricted movement. The aortic root diameter was 56mm upon CT scan evaluation. The patient underwent cardiac catheterization and Swan–Ganz catheter placement, followed by aortic valve and aortic root replacement with a composite graft (via the modified Bentall and DeBono technique). To facilitate this procedure during cardiopulmonary bypass, a 3 mg kg^–1^ bolus of heparin was administered. Immediately following the performance of extracorporeal circulation, the pulmonary pressure curve disappeared and, for this reason, the Swan–Ganz catheter was removed. Suddenly, the patient experienced massive haemoptysis. The patient’s PaO_2_ levels decreased rapidly and bronchial bleeding could be stopped only after protamine infusion and platelet transfusion. A bronchoscopy was essential for the removal of clots from both branches of the bronchi, and this procedure was able to restore sufficient blood saturation. No thoracic bleeding was found. When the chest radiograph was performed in the intensive care unit, a right basal pleural effusion was observed (Figure 1). The patient recovered well after 3 days of mechanical ventilation owing to a very low PaO_2_ level. 1 week later, before discharge, the patient underwent CT angiography, which revealed the presence of a pulmonary PSA (Figure 2).

## Treatment and outcomes

**Figure 3.**
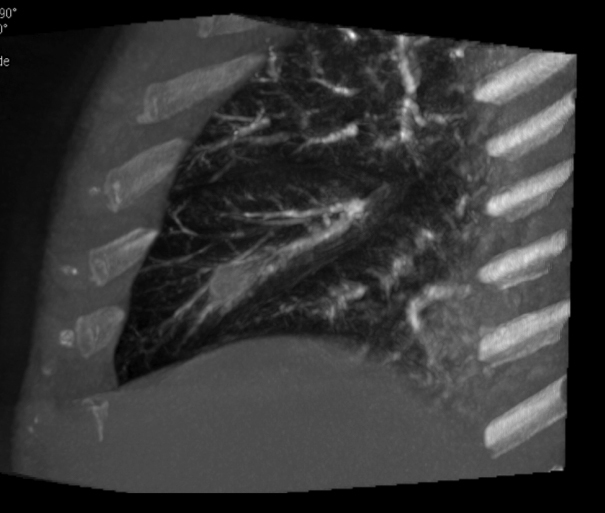
Coronal view of cone-beam CT angiography documenting the pseudoaneurysm.

**Figure 4.**
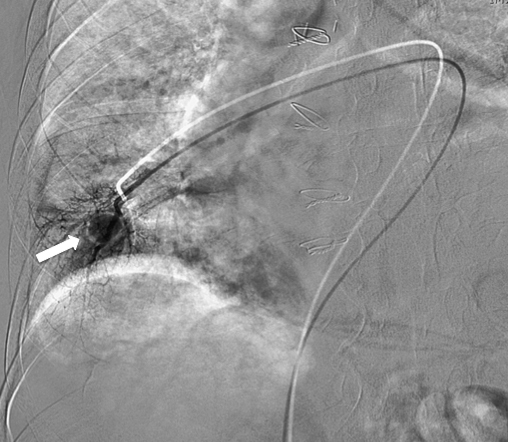
Angiography following selective catheterization of the medial segment branch of the right middle lobe pulmonary artery showing the pseudoaneurysm (arrow).

**Figure 5.**
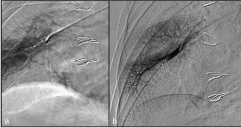
The angiogram performed following plug deployment documented persistent filling of the pseudoaneurysm (a); the angiogram performed after embolization with N-butyl cyanoacrylate revealed complete embolization (b).

The case was presented to our interventional radiology team, and an angiogram was performed. Using the right common femoral vein approach, a 7 French sheath was deployed; a 100-cm vertebral-shaped catheter (Cordis, Miami Lakes, FL) was introduced into the right PA and an angiogram was performed. The first angiogram did not reveal the PSA. CBCT angiography (Allura Xper FD20 Philips, Best, The Netherlands) revealed the PSA and helped the operators to catheterize the correct branch of the right PA (Figure 3). Specifically, following catheterization of the medial segment branch of the right middle lobe PA, the PSA was identified (Figure 4). A 4-mm AVP IV (AGA Medical Corp., Plymouth, MN) was deployed. Several angiograms were performed within the next 10 min; they revealed persistent filling of the PSA. Superselective catheterization was undertaken with a 2.7 French coaxial microcatheter (Progreat, Terumo, Tokyo, Japan); 0.2 ml of Glubran II (GEM, Viareggio, Italy) was injected. The angiogram was performed immediately following this procedure and it confirmed complete embolization (Figure 5). The patient remained asymptomatic and was discharged 2 days later.

## Discussion

The placement of a Swan–Ganz catheter into the PA can cause different complications, such as inadvertent arterial puncture (1.9%), pneumothorax (0.5%), arrhythmias (12.5% to over 70%), catheter-related bacteraemia (1.3%–2.3%), sepsis, PA thrombosis and rupture.^6^ PA rupture is a serious complication that has been observed with the use of Swan–Ganz catheters; it has an estimated incidence of 0.031% and 0.05%, and mortality rates of 50%–70% secondary to aspiration and asphyxia following intrapulmonary haemorrhage or exsanguination.^6^ After PA rupture, the sequelae include one of the following: intrapulmonary haemorrhage, the formation of a PSA or re-endothelialization of the injury.

The primary symptom of PAPs is haemoptysis, which is often massive.^3^ A bronchoscopy or CT scan is usually performed to locate the haemorrhage in a specific lobe; it is this knowledge that enables selective angiographic evaluation.^5^

In patients who survive the initial haemoptysis resulting from a PA rupture, the formation of a PSA has reportedly occurred anywhere between several minutes to 7 months later.^7^ The incidence of recurrent bleeding from the PSA is estimated at 30–40%, with a mortality rate of 40–70%.^6^

A number of risk factors have been implicated in the development of PA rupture; these include systemic anticoagulation, PA hypertension, long-term steroid therapy, surgically induced hypothermia, cardiac decompression and manipulation during surgery, being older than 60 years of age, and being female.^8^

The treatment options for PAP include positive end-expiratory pressure, an emergency thoracotomy for PA ligation, segmentectomy or lobectomy, and transcatheter embolization.^7,8^

As reported by Nellaiyappan et al^9^ in their review of the literature, coil embolization appears to be the preferred method when treating a PSA, with a technical success rate of 89% in the reviewed patient population.

To the best of our knowledge, only three studies employed the AVP as an embolic agent.^10–12^ There are no guidelines available to help determine which embolic material to use; the final decision is often made based on the operator’s experience and his or her level of confidence. The efficacy of embolization depends on a combination of different factors, such as the location of the bleed site, the patient’s clot-forming ability, and local vasospasm. The most commonly used embolic materials include 0.018-inch pushable microcoils. Microcoils are permanent embolic agents; the fibers of the coils induce clot formation. Plugs are built with nitinol braids, which are able to self-expand. The small holes in the plug reduce the blood flow and form a clot to seal the device. Therefore, successful occlusion following deployment of the AVP is not immediate, but requires a few minutes. Conversely, occlusion is never achieved in patients with coagulopathy or low platelet counts.^13^

We presented a case of a patient receiving anticoagulation therapy for a recent heart surgery with an absolute contraindication to its suspension. After the deployment of a 4-mm AVP, the PA remained patent until the angiogram was performed 10 min later (Figure 5a). NBCA has a great haemostatic effect, which does not depend on the patient’s clot-forming ability. This represents an advantage when treating anticoagulated patients. Another advantage is the low cost of the glue that is used. The major disadvantage is that it is difficult to penetrate the vasculature, which requires considerable experience. A total of 0.2 ml of Glubran II was resolutive; complete embolization was ultimately achieved (Figure 5b).

Several studies confirmed the role of embolization in anticoagulated patients.^14^ In patients receiving anticoagulant and/or antiplatelet therapy, the time to occlusion after the deployment of an embolic agent could not be standardized; either time to occlusion was prolonged, or complete embolization could not be achieved. Among permanent embolic agents, mechanical agents fill the vessel space; moreover, some of these agents feature intrinsic thrombogenic properties. Similarly, AVP is a foreign body, and its small holes reduce blood flow and form a clot to seal the device. There may be persistent patency without the application of further embolic material in uncoagulated patients.^13,14^ In our opinion, this is what likely occurred with our patient. NBCA, like ethylene–vinyl alcohol copolymer (Onyx Liquid Embolic System; ev3 Neurovascular, Irvine, CA), presents with a great haemostatic effect and a low recurrent bleeding rate, independent of coagulation status.^15^

The case presented here suggests that a highly haemostatic polymeric agent could be used as the first choice of treatment in similar situations, as it is able to achieve rapid and effective occlusion.

## Learning points

1. PAP should be taken into consideration in patients with haemoptysis following Swan–Ganz catheter deployment.
2. Percutaneous embolization is a safe and effective treatment option.
3. In anticoagulated patients, embolization with highly haemostatic polymeric agents could be the first choice of treatment, as it achieves rapid and effective occlusion.
